# Frontiers in cadmium mitigation: harnessing Nitrate Transporter 1 (NRT1) for plant systems

**DOI:** 10.1007/s00425-026-04963-7

**Published:** 2026-03-04

**Authors:** Deyvid Novaes Marques, Ricardo Antunes Azevedo

**Affiliations:** Department of Genetics, Luiz de Queiroz College of Agriculture (ESALQ), University of São Paulo (USP), Piracicaba, São Paulo, SP 13418-900 Brazil

**Keywords:** Cadmium stress, Molecular biology, NRT1 transporters, Nitrogen, Plant biotechnology, Plant physiology

## Abstract

**Main conclusions:**

Within this still-developing research landscape, NRT1 transporters are increasingly recognized as regulators of nitrate reallocation and related cadmium (Cd) responses in plants, integrating transporter activity, hormonal signaling, and gene regulation.

**Abstract:**

Cadmium (Cd) is a toxic heavy metal and a major environmental pollutant that acts as a significant abiotic stress factor in plant systems. Its contamination poses a persistent threat to both ecosystems and food safety, with important implications for phytoremediation and broader environmental management strategies. In this article, we present a perspective on the role of the Nitrate Transporter 1 (NRT1) family in plant Cd research, a topic that warrants further investigation given that emerging evidence has linked NRT1 members to Cd tolerance and to the modulation of Cd uptake and accumulation in model species, crop plants of food safety importance, and plant species with relevance to Cd management research. Although functional studies remain relatively limited, current evidence suggests and highlights that specific NRT1 isoforms influence Cd distribution and plant growth under stress conditions. In addition, hormonal regulation, genetic engineering, and emerging biotechnological tools provide opportunities to fine-tune NRT1 activity. We also outline key priorities for future research. Overall, this perspective offers a forward-looking view on leveraging NRT1 transporters and related genes for biological engineering strategies aimed at improving plant performance and food safety in Cd-contaminated environments, while contributing to broader Cd mitigation efforts.

**Supplementary Information:**

The online version contains supplementary material available at 10.1007/s00425-026-04963-7.

## NRT1 transporters in the context of cadmium exposure in plants

The escalating environmental contamination with cadmium (Cd) poses a significant and persistent threat to various agricultural and plant systems. In this context, we have extensively explored insights related to plant Cd research (Marques et al. 2023, 2025a, b; Marques and Mason 2025; and references therein). Nitrate Transporter 1 (NRT1) transporters have emerged as key integrators of nutrient and heavy metal homeostasis in plants, coordinating nitrate allocation with stress responses to metals such as Cd. It has been highlighted that NRT1 is a plasma membrane protein involved in nitrate uptake in roots as well as in nitrate sensing and signal transduction. Beyond these functions, NRT1 proteins and their associated gene expression influence broader physiological processes, integrating nitrogen acquisition with hormonal signaling and gene regulatory networks across multiple plant species under diverse environmental conditions (Cheng et al. 2025; Shi et al. 2025; Wang et al. 2025a, b; Morales de Los Ríos et al. 2026). Together, the available evidence indicates that NRT1 transporters function not merely as nutrient carriers, but as integrative regulators capable of linking nitrogen metabolism to stress responses across multiple plant systems. Therefore, compared with metal-specific transporters, NRT1 emerges as a strategic integrator of nutrient signaling and stress adaptation, providing a mechanistic framework that might extend beyond direct Cd transport. Thus, previously recognized for their crucial roles in nitrate uptake and signaling, these versatile transporters are now increasingly implicated in the modulation of plant responses to Cd toxicity. Here, we offer a perspective on the contribution of NRT1 transporters and related genes in the context of plant Cd exposure, an area that merits deeper investigation as a growing body of evidence associates NRT1 members with modulated Cd tolerance and altered Cd accumulation patterns across plant model systems, food-relevant crops, and other species involved in Cd management research.

In Table 1, representative evidence highlighting the roles of specific NRT1 isoforms across distinct plant systems is compiled. Complementing these functional aspects, Table 2 outlines key experimental approaches that have been employed to investigate the involvement of NRT1 transporters and related genes under Cd exposure conditions.

**Table 1:** Examples of aspects regarding functional roles of NRT1 isoforms and related genes in Cd regulation and mitigation across plant species

Isoform	Function and species context	Citation
NRT1.1	Mediates Cd uptake and tolerance by regulating nitrate allocation to roots; its inhibition reduces Cd accumulation while its presence under Cd stress induces signaling pathways (e.g., *NRG2*) to modulate aspects involving other transporters like NRT1.5 and NRT1.8 (*Arabidopsis thaliana*)	Mao et al. (2014), Jian et al. (2019), Guan et al. (2021)
NRT1.2/AIT1	Functions as a root ABA importer; its activity triggers the downregulation of metal transporters-related genes (e.g., *IRT1*) to inhibit Cd entry or enhances the efficacy of ABA-mediated Cd mitigation in contaminated soils (*A. thaliana*; *Brassica chinensis*)	Pan et al. (2020), Wang et al. (2024)
NRT1.5	Involved in nitrate distribution; it is typically downregulated by Cd stress or ABA signaling to promote nitrate accumulation in roots, which enhances Cd tolerance (*A. thaliana*; *Brassica napus*)	Chen et al. (2012), Wang et al. (2018), Zhang et al. (2014)
NRT1.8	Facilitates nitrate removal from xylem and reallocation to roots under Cd stress; it is upregulated by Cd and ET/JA signaling to maintain root tip growth and promote cell proliferation (*A. thaliana*)	Li et al. (2010), Zhang et al. (2014), Wang et al. (2026)
NRT1 (General/Family)	Exhibits varied roles including downregulation under stress to decrease nitrate uptake or stabilization by nanoparticles (ZnO NPs) via m^6^A methylation to enhance Cd resistance (*Hibiscus cannabinus*; *Vigna radiata*; *Glycine max*)	Chen et al. (2021), Leng et al. (2024), Wang and Fang (2024)

*ABA* abscisic acid, *Cd* cadmium, *EIL1* EIN3-LIKE 1, *EIN3* Ethylene Insensitive 3, *ET* ethylene, *IRT1* Iron-Regulated Transporter 1, *JA* jasmonic acid, *m*^*6*^*A* N6-methyladenosine, *NRG2* Nitrate Regulatory Gene, *NRT1* Nitrate Transporter 1, *ZnO NPs* zinc oxide nanoparticles

**Table 2:** Examples of experimental approaches and related research aspects highlighting the role of NRT1 transporters and related genes under Cd exposure conditions

Experimental approach	Exemplary key findings and related research aspects	Citation
Characterization of *A. thaliana nrt1.5* mutants under Cd stress through hydroponic growth, gene expression analysis, and measurement of ion and metabolite levels	Functional disruption of *NRT1.5* enhanced tolerance to Cd stress, and *NRT1.5* expression was downregulated by Cd treatment, leading to increased nitrate and Cd accumulation in roots	Chen et al. (2012)
Comparative transcriptomic analysis of two kenaf cultivars (GH-tolerant, YJ-sensitive) under Cd stress (10 mg L^−1^ CdCl_2_) for 7 days, including gene expression analysis, which identified NRT1 as differentially expressed	A nitrate transporter gene (*NRT1*; KN48595_c0_g1) was found to be downregulated in both kenaf cultivars under Cd stress (0.36-fold in GH and 0.18-fold in YJ), suggesting a possible decrease in nitrate uptake under these conditions	Chen et al. (2021)
Hydroponic culture of *A. thaliana* wild-type (Col-0, Ler) and *nrt1.1*, *nrt2.1–2*, *nrt2.2*, and *nrt2.4* mutants under low-nitrate conditions with Cd treatment, measuring Cd and nitrate concentrations, uptake rates (including 15N), and gene expression of nitrate transporters	Loss of function of NRT1.1 and NRT2.1 reduced Cd accumulation; NRT2.1 contributed more to Cd uptake than NRT1.1; Cd accumulation controlled by HATS was positively correlated with nitrate uptake; nitrate supply increased Cd uptake under low nitrate; Cd stress impaired nitrate uptake by reducing *NRT* gene expression	Guan et al. (2021)
Characterization of *A. thaliana* wild-type (Col-0) and *nrt1.1* mutants (chl1-1, chl1-5, chl1-13) under Cd (Cd^2+^) stress (20 µM CdCl_2_) through hydroponic growth, measurement of biomass, chlorophyll, proline, MDA, SOD, Cd^2+^ and nitrate concentrations, 15N tracer assays, photosynthetic parameters, gene expression analysis of *NRT1.5* and *NRT1.8*, V-ATPase and V-PPase activities, and Cd^2+^ and nitrate levels in protoplasts and vacuoles	NRT1.1 regulates nitrate allocation to roots under Cd^2+^ stress, which contributes to Cd^2+^ tolerance in *A. thaliana*; Cd^2+^ stress induced *NRG2* expression in wild-type, but not in nrt1.1 mutants; NRG2 functions downstream of NRT1.1 in regulating nitrate allocation by modulating the expression of NRT1.5 and NRT1.8; Cd^2+^ stress enhanced Cd^2+^ and nitrate accumulation in root vacuoles, facilitated by increased V-ATPase and V-PPase activities in the wild-type but not in *nrt1.1* mutants	Jian et al. (2019)
Transcriptome analysis of mung bean (*Vigna radiate*) seedlings under Cd stress with and without foliar application of ABA over 9 days in different tissues	Upregulation of *NRT1/PTR* family protein encoding genes was observed mainly in leaves and stems of mung bean seedlings treated with ABA under Cd stress, suggesting a potential role in nutrient and/or Cd transport	Leng et al. (2024)
Functional characterization of *A. thaliana* NRT1.8 through gene expression analysis under nitrate and Cd stress, protein localization, nitrate uptake assays in *Xenopus* oocytes, and physiological studies of *nrt1.8* mutants under Cd and varying nitrate conditions, also comparing its regulation with *NRT1.5* under stress	*NRT1.8* encodes a low-affinity nitrate transporter located in xylem parenchyma cells, responsible for nitrate removal from xylem sap; *NRT1.8* expression is upregulated by Cd stress in roots; the *nrt1.8* mutant exhibits nitrate-dependent Cd sensitivity and altered nitrate distribution with less nitrate in roots under Cd stress; *NRT1.5* and *NRT1.8* show opposite regulation under Cd and other environmental stresses	Li et al. (2010)
Compared *A. thaliana* wild-type (Col-0, Ler) and *nrt1.1* mutants (*chl1.5, nrt1.1-1, chl1.6*) under Cd stress in hydroponic culture, measuring nitrate uptake (using 15N), Cd accumulation, biomass, and gene expression of nitrate transporters like NRT1.1, including split-root experiments and treatment rotation systems to investigate the relationship between nitrate and Cd uptake	Inhibition of NRT1.1 activity by Cd reduced nitrate uptake, which consequently decreased Cd uptake in *A. thaliana*, leading to enhanced Cd tolerance in *nrt1.1* mutants in nitrate-containing medium; this effect was dependent on nitrate uptake as no difference in Cd levels was observed in nitrate-free medium between wild-type and *nrt1.1* mutants; NRT1.1 might regulate the uptake of Cd and other cations through a common mechanism requiring simultaneous nitrate uptake	Mao et al. (2014)
Characterization of *A. thaliana* wild-type (Col-0), *ait1* mutants (*AIT1/NRT1.2* deficient), *AIT1*-overexpressing transgenic lines, and *irt1* mutants under Cd stress (10 μM) with or without exogenous ABA (0.5 μM) in hydroponic culture. Measurements included biomass, physiological parameters (e.g., NPQ), Cd and endogenous ABA concentrations in roots and shoots, and RT-qPCR expression analysis of *AIT1* and metal transporters-related genes (*IRT1*, *ZIP1*, *ZIP4*, *Nramp1*), alongside pharmacological assays using *IRT1* inhibitor	AIT1 (NRT1.2) functions as a critical root ABA importer that mediates Cd mitigation; exogenous ABA significantly reduced Cd accumulation in wild-type shoots and *AIT1*-overexpressing lines, whereas these effects were attenuated in *ait1* mutants. Mechanistically, AIT1-mediated ABA import triggers the downregulation of the metal transporter-related *IRT1* in roots, thereby inhibiting Cd entry and alleviating Cd-induced growth inhibition and photosynthetic damage	Pan et al. (2020)
Vegetable soybean seedlings (Zhenong 6) were subjected to Cd (Cd) stress with or without ZnO NPs in hydroponic solution. m^6^A methylome analysis and RNA-seq were performed on root samples to identify DEGs with differential m^6^A modification. Plant m^6^A Editors (PMEs) were used to specifically reduce the m^6^A level of *NRT1* in transgenic plants, and their Cd tolerance was evaluated. SELECT-PCR and qPCR were used to analyze m^6^A levels and gene expression. Hairy root transformation was performed to generate transgenic lines	ZnO NPs treatment reduced the m^6^A level and stabilized the mRNA of NRT1. Transgenic plants with reduced m^6^A levels of *NRT1* showed increased expression of *NRT1* and stronger Cd tolerance compared to control plants. This suggests that ZnO NPs enhance Cd tolerance in vegetable soybean by regulating the m^6^A methylation level of stress-responsive genes such as *NRT1*, which affects its expression. NRT1 plays a positive role in resistance to Cd^2+^ stress	Wang and Fang (2024)
Screening of 18 pak choi (*Brassica chinensis* L.) varieties for *NRT1.2* expression intensity, followed by the selection of low (Aidi), medium (Libai 1), and high (Baisuzhen 207Q) expression genotypes. These were grown in three types of contaminated soils (Zhejiang, Sichuan, Heilongjiang) under the synergistic treatment of the ABA-generating bacterium *Azospirillum brasilense* and corn straw biochar. Analysis focused on biomass, endogenous ABA levels, and the accumulation of heavy metals (Cd, Ni, Pb, Zn) in shoots and roots	High *NRT1.2* expression intensity significantly enhances the efficacy of the bacterial-biochar synergy in mitigating Cd stress. In high-expression genotypes, the treatment reduced shoot Cd accumulation and increased fresh weight. The findings demonstrate that elevated *NRT1.2* expression facilitates increased cellular uptake of bacteria-derived ABA, which might trigger the regulation of metal-transport genes, while biochar supports microbial colonization and reduces soil metal bioavailability	Wang et al. (2024)
Hydroponic culture of *A. thaliana* wild-type and ABA mutants under Cd stress with/without ABA, and *Brassica napus* seedlings under Cd stress with/without exogenous ABA; measuring physiological parameters (chlorophyll, proline, MDA, ABA, nitrate, Cd in root vacuoles/protoplasts), gene expression of *NRT1.5* and *NRT1.8*, and proton pump activity (V-ATPase, V-PPase) in *A. thaliana*	*NRT1.5* expression is downregulated by ABA signaling under Cd stress, promoting nitrate accumulation in roots of both *A. thaliana* and *Brassica napus*, while *NRT1.8* does not respond to ABA; higher proton pump activity in *A. thaliana* wild-type enhances vacuolar nitrate and Cd accumulation, reducing cytoplasmic Cd and increasing Cd resistance; exogenous ABA inhibits Cd absorption in *Brassica napus*, enhancing its resistance	Wang et al. (2018)
Investigation of *NRT1.8* spatio-temporal expression and polar localization in *A. thaliana* root tips under Cd stress and high nitrate, including NMT analysis of nitrate fluxes, root meristem cell counting, and transcriptome analysis of root tips in *nrt1.8* mutants	NRT1.8 shows polar localization toward the soil side in epidermal cells; Cd and high nitrate induce NRT1.8 to uptake nitrate from LRC into inner cells, maintaining NO levels in root tips; this mechanism antagonizes root meristem growth inhibition and promotes cell proliferation under Cd stress	Wang et al. (2026)
Characterization of *A. thaliana* wild-type and mutants of ET/JA signaling (e.g., *ein2-50*, *coi1-1*, *ein3 eil1*) and nitrate transporters (*nrt1.5*, *nrt1.8*) under Cd stress (200 µM CdCl_2_). The study utilized gene expression analysis (RT-qPCR), protein–DNA interaction assays (EMSA and ChIP), 15N tracer assays, and physiological measurements of root growth	Cd stress activates an ET/JA signaling module that coordinates SINAR. This module converges at EIN3/EIL1, which directly downregulates *NRT1.5* while inducing ERFs, such as ORA59, to upregulate *NRT1.8*. This highly coordinated regulation facilitates nitrate reallocation to roots, enhancing Cd tolerance. This adaptive response mediates the trade-off between environmental adaptation and plant growth in a nitrate reductase-dependent manner	Zhang et al. (2014)

*15N* nitrogen-15, *ACC* 1-aminocyclopropane-1-carboxylic acid, *ABA* abscisic acid, *ChIP* chromatin immunoprecipitation, *Cd* cadmium, *Cd*^*2+*^ divalent cadmium cation, *DEGs* differentially expressed genes, *EMSA* electrophoretic mobility shift assay, *ERFs* ethylene response factors, *EIL1* EIN3-LIKE 1, *EIN3* Ethylene Insensitive 3, *ET* ethylene, *JA* jasmonic acid, *HATS* high-affinity nitrate transporters, *LRC* lateral root cap, *MDA* malondialdehyde, *m*^*6*^*A* N6-methyladenosine, *NO* nitric oxide, *NPs* nanoparticles, *NPQ* non-photochemical quenching, *NRG2* Nitrate Regulatory Gene, *NMT* Non-invasive Micro-test Technology, *NRT* nitrate transporter, *NRT1.1* nitrate transporter 1.1, *NRT1.5* nitrate transporter 1.5, *NRT1.8* nitrate transporter 1.8, *NRT2.1* nitrate transporter 2.1, *NRT2.2* nitrate transporter 2.2, *NRT2.4* nitrate transporter 2.4, *PMEs* plant m^6^A editors, *PTR* peptide transporter, *ORA59* Octadecanoid-Responsive *Arabidopsis* AP2/ERF59, *SOD* superoxide dismutase, *SINAR* stress-initiated nitrate allocation to roots, *V-ATPase* ATP-dependent proton pump, *V-PPase* pyrophosphate-dependent proton pump

## Toward a framework investigating NRT1 isoforms and cadmium exposure in plants

Although studies examining NRT1 across multiple plant systems are still evolving, the understanding that NRT1 and Cd exposure are interconnected provided the groundwork for investigating the roles of such transporters beyond their primary function in nitrogen management. Some authors conducted a functional characterization of NRT1.8, a member of the NRT1 nitrate transporter family in *Arabidopsis thaliana*. They showed that NRT1.8 mediates nitrate removal from the xylem sap, as evidenced by its predominant expression in xylem parenchyma cells, plasma membrane localization, and low-affinity nitrate uptake activity demonstrated in electrophysiological assays using *Xenopus laevis* oocytes and uptake assays. Importantly, disruption of NRT1.8 markedly increased nitrate concentration in the xylem sap, directly supporting its role in xylem nitrate unloading. The study also highlighted that *NRT1.8* is strongly upregulated by Cd^2⁺^ stress in roots, uniquely among genes involved in nitrate assimilation (Li et al. 2010). Consequently, the *nrt1.8-1* mutant displayed a nitrate-dependent Cd^2⁺^-sensitive phenotype, associated with a reduced proportion of nitrate allocated to roots under Cd^2⁺^ stress relative to wild-type plants. These findings indicated that NRT1.8-mediated nitrate distribution is important for Cd^2⁺^ tolerance (Li et al. 2010), likely by influencing nitrate partitioning between roots and shoots under Cd stress and modulating downstream stress response pathways.

Chen et al. (2012) in turn studied NRT1.5 as an important component of plant responses to multiple abiotic stresses, including Cd. Their study demonstrated that NRT1.5, a nitrate transporter responsible for xylem loading in roots, is transcriptionally downregulated under Cd stress. Functional disruption of *NRT1.5* enhanced tolerance to Cd stress and was accompanied by the upregulation of *NRT1.8* in *nrt1.5* mutant plants (Chen et al. 2012). The coordinated, but functionally distinct, regulation of *NRT1.5* and *NRT1.8* under Cd stress was reported by Li et al. (2010). Altogether, this set of information may support the hypothesis that active nitrate reallocation to roots represents a common adaptive response to Cd exposure.

Mao et al. (2014) showed that Cd inhibits NRT1.1-mediated nitrate uptake, and that suppressing this process reduces Cd accumulation in *A. thaliana* roots in a nitrate-dependent manner. In nitrate-containing medium, loss of NRT1.1 functions not only lowered Cd and other metal levels, but also improved biomass production under Cd stress. These findings indicate that reduced nitrate uptake can simultaneously enhance tolerance and growth in contaminated soils, while also suggesting that NRT1.1 may influence the transport of Cd and other cations. Although this inhibition may affect nutrition-related aspects depending on the plant system used, targeted modification of NRT1.1 activity in crops or other plant species might represent a strategy to limit Cd accumulation in edible tissues while sustaining biomass, offering a potential avenue to improve both food safety and plant productivity through biological engineering. However, the potential impacts on the uptake of essential nutrient cations should also be carefully evaluated.

More recent studies in *A. thaliana* have provided deeper insight into the regulatory mechanisms governing *NRT1.1* function under Cd stress. Jian et al. (2019) demonstrated that *NRT1.1* regulates Cd stress-induced nitrate allocation to roots through a signaling cascade involving *Nitrate Regulatory Gene 2 (NRG2)*, which functions downstream of *NRT1.1*. Under Cd exposure, repression of *NRT1.5* and induction of *NRT1.8* promoted increased nitrate allocation to roots and elevated root-to-shoot nitrate ratios. Importantly, total Cd uptake did not differ between Col-0 and the *nrt1.1* mutant; instead, *NRT1.1* was associated with the preferential allocation of Cd^2⁺^ and nitrate to root vacuoles, thereby reducing cytosolic Cd toxicity and limiting Cd translocation to the shoots (Jian et al. 2019). These findings refine previous views of *NRT1.1* function by highlighting its role in regulating nitrate partitioning and intracellular Cd sequestration, rather than directly mediating Cd uptake, and underscore the complexity of engineering Cd tolerance without disrupting nitrate nutrition.

The role of hormonal signaling in modulating the Cd stress response extends to the regulation of transporters, which includes abscisic acid (ABA)-dependent signaling pathways. Based on experimental evidence and related research perspectives, Wang et al. (2018) proposed a model in which Cd exposure induces ABA signaling, leading to the repression of *NRT1.5* expression without affecting *NRT1.8*, promoting nitrate retention in roots and reducing nitrogen use efficiency while enhancing Cd tolerance in *A. thaliana*. Cd stress stimulates proton pump activity in roots, promoting root accumulation and vacuolar sequestration of both nitrate and Cd, while limiting cytosolic Cd levels and enhancing nitrate allocation to the roots. A similar ABA-dependent regulation was observed in *Brassica napus*, where *BnNRT1.5*, but not *BnNRT1.8*, responded to ABA, contributing to improved Cd resistance along with hindered Cd absorption (Wang et al. 2018). This adds a hormonal signaling dimension to NRT1-mediated Cd responses and highlights the role of proton pump-driven electrochemical gradients and energetic constraints in plant adaptation to combined nutrient and Cd contexts, while also suggesting potential targets for manipulating these transporters.

More recently, within the framework of ABA research, Leng et al. (2024) reported that foliar application of ABA on mung bean (*Vigna radiata*) seedlings under Cd stress significantly modulated the expression of transporter-related genes, including members of the NRT1/PTR (Nitrate Transporter 1/Peptide Transporter), displaying organ-specific differential induction patterns (Leng et al. 2024). While ABA’s primary role in this study was linked to mitigating general Cd toxicity, the modulation of NRT1/PTR family genes points to a potential indirect role in Cd handling or tolerance through nutrient and hormone-associated transport systems. Leng et al. (2024) also observed that elevated ABA levels potentially promoted the activation of ABA receptors such as PYLs and modulated hormone-related genes in leaves and stem, reinforcing stress-adaptive signaling pathways that intersect with nutrient regulation.

Other studies have investigated the NRT1.2 isoform (also known as AIT1 or NPF4.6) as a relevant hub for mediating Cd tolerance through its dual role as a nitrate and ABA importer. Specifically, Pan et al. (2020) provided molecular evidence for this mechanism in *A. thaliana* by demonstrating that while exogenous ABA reduced Cd levels in wild-type shoots and roots, these effects were significantly enhanced in *AIT1*-overexpressing plants. Conversely, *ait1* mutants exhibited significantly higher Cd accumulation compared to overexpressing lines. This research focused on a molecular signaling pathway where AIT1-mediated ABA import into root cells triggers the downregulation of the metal transporter gene *IRT1* (Iron-Regulated Transporter 1) (Pan et al. 2020), thereby inhibiting a route for Cd entry into the plant. Expanding these findings to an agronomic context, Wang et al. (2024) investigated the interplay between transport capacity and soil amendments using *Brassica chinensis* (pak choi) genotypes selected for varying *NRT1.2* expression intensities. The study revealed a potent synergy between biochar and the ABA-generating bacterium *Azospirillum brasilense*, which was most effective in genotypes with high natural *NRT1.2* expression. In these high-expression varieties, the synergistic treatment reduced Cd accumulation in the shoots (part of a broader heavy metal reduction) and increased fresh weight (Wang et al. 2024). These studies highlight an integrated strategy where the plant model-related insights along with the effectiveness of microbial and soil amendments for Cd mitigation are maximized by selecting crop materials with elevated *NRT1.2* expression, which might facilitate higher cellular ABA uptake and subsequent modulation of metal-transport genes. Notably, given IRT1’s dual role in mediating iron uptake and modulating Cd entry, careful regulation of *IRT1* expression is essential. Manipulations that enhance ABA-mediated Cd tolerance via NRT1.2 may inadvertently modulate iron homeostasis, highlighting the need for balanced strategies when targeting metal transporters in crops.

Although the mentioned investigations report ABA-associated modulation of NRT1-related genes, the molecular framework linking these transporters to coordinated nitrogen-responsive transcriptional control remains incompletely resolved. In this context, insights from broader nitrogen and plant signaling research provide a mechanistic basis for understanding how ABA and nitrate signaling may converge within the plant cell-related context. For example, in *A. thaliana*, the main conjugated storage form of ABA is ABA–glucose ester (ABA-GE). This inactive conjugate accumulates predominantly in the vacuole and is also detected in the xylem sap and possibly in the apoplastic space. The mobilization of active ABA relies on β-glucosidases, including BGLU10, which operates in the vacuole and hydrolyzes ABA-GE, releasing free ABA to support stress-responsive signaling (reviewed by Wang et al. 2018). Efficient vacuolar compartmentalization of toxic metals and excess nutrients relies on the energization of the tonoplast membrane. This process is mediated by the proton-translocating activities of V-ATPase and V-PPase, which pump protons into the vacuolar lumen and generate a proton motive force. The resulting electrochemical gradient provides the driving force for secondary transport systems that mediate the accumulation of Cd and nitrate inside the vacuole, thereby contributing to cytosolic detoxification and ion homeostasis related to ABA signaling (Wang et al. 2018). In turn, the NIN-like transcription factor NLP4—known to recognize nitrate-responsive *cis*-regulatory elements (NREs) in gene promoters and regulate nitrate-dependent transcription—acts as a central hub in ABA signaling. Triggered by ABA perception via a NRT1 transporter/receptor family at the plasma membrane, NLP4 shows pronounced nuclear enrichment and orchestrates a substantial fraction of ABA-responsive genes through hierarchical transcriptional cascades, thereby integrating hormonal and nitrogen-related signaling pathways (Ma et al. 2025). In addition, ABA accumulation under unfavorable nitrogen-related conditions suppresses ABI1/ABI2 phosphatases, sustaining CIPK15 and CIPK23 kinase activity toward shared nitrogen transporter targets, further illustrating the convergence between ABA signaling and nitrogen transport regulation (Rivero-Marcos 2025). These findings underscore that ABA signaling under stress conditions is closely coordinated with nitrogen-responsive regulatory networks, operating through both plasma membrane-associated components and nuclear transcriptional regulators. Overall, however, the specific involvement of different NRT1 isoforms in this integrated hormonal-nitrogen signaling framework still requires further investigation, particularly within the context of Cd exposure in multiple plant species in addition to plant models and the functional specialization of distinct NRT1 members.

Expanding the hormone-related NRT1 research context, in *A. thaliana*, Cd stress activates a specific hormonal signaling network where ethylene (ET) and jasmonic acid (JA) pathways converge to coordinate Stress-Initiated Nitrate Allocation to Roots (SINAR). This process is governed by a molecular module centered on the transcription factors EIN3 and EIL1, which integrate signals from both ET and JA cascades. Mechanistically, EIN3 directly binds to the promoter of *NRT1.5* to suppress its expression, thereby reducing nitrate loading into the xylem. Simultaneously, EIN3/EIL1 (Ethylene Insensitive 3/EIN3-LIKE1) induce the expression of ethylene response factors (ERFs), specifically ORA59 (Octadecanoid-Responsive *Arabidopsis* AP2/ERF59), ERF1B (Ethylene Response Factor 1B), and ERF104 (Ethylene Response Factor 104). These ethylene response factors (ERFs) bind to GCC boxes in the *NRT1.8* promoter to drive its upregulation, facilitating nitrate unloading from the xylem sap back into root tissues. This reciprocal regulation enhances Cd tolerance but results in a physiological trade-off, as the increased allocation of nitrate to roots significantly reduces plant growth under non-stressed conditions. Notably, the functional impact of SINAR on both stress tolerance and growth is strictly dependent on nitrate reductase activity (Zhang et al. 2014).

Beyond the confirmed EIN3/EIL1-ERF circuit, it is suggested that alternative signaling pathways, such as ABA, may provide additional, though subtle, contributions to the coordinated regulation of *NRT1.5* and *NRT1.8* under Cd stress. Furthermore, because ET and JA are produced under a wide range of unfavorable conditions, the ET/JA-NRT signaling module is proposed to represent a universal adaptive mechanism for plants to respond to diverse environmental stresses beyond heavy metals. Regarding the signaling molecule involved in the SINAR-mediated growth balance, while nitrate itself is a likely candidate, it is speculated that nitrogen assimilates or derivatives, such as glutamate (Glu) or glutamine (Gln), might also function as signal molecules perceived by the plant to mediate these responses (Zhang et al. 2014).

In a broader transcriptomic approach, Chen et al. (2021) investigated the responses of two kenaf (*Hibiscus cannabinus* L.) cultivars with contrasting Cd tolerance, revealing that *NRT1* was identified as a critical Cd-induced differentially expressed gene. This finding in a plant known for its high heavy metal tolerance and accumulation potential suggests a conserved involvement of these transporters on the Cd stress response across diverse plant species. The observed modulation of specific NRT1 isoforms in kenaf might offer promising targets for enhancing its phytoremediation efficiency.

A particularly innovative strategy for targeting NRT1-related mechanisms to modulate Cd tolerance was reported by Wang and Fang (2024), who employed targeted mRNA demethylation using Plant m^6^A Editors (PMEs)—engineered RNA-editing components that allow targeted modulation of N6-methyladenosine (m^6^A) marks on RNA—to specifically reduce m6A levels on *NRT1* transcripts in soybean (*Glycine max*). This reduction in m^6^A methylation increased *NRT1* mRNA stability and expression, and transgenic plants carrying PMEs targeting *NRT1* exhibited enhanced Cd tolerance under ZnO nanoparticle treatment compared with controls (Wang and Fang 2024). These findings identify *NRT1* as a stress-responsive gene subject to post-transcriptional regulation via m^6^A modification and highlight PMEs as a promising tool for targeted gene manipulation to improve Cd tolerance in crops. However, the specific *NRT1* isoforms involved and their precise roles in Cd-related process warrant further investigation in the crop research scenario.

Recent evidence indicates that m^6^A-mediated regulation of Cd responses is unlikely to represent a legume-specific adaptation and instead appears to be part of a broader, evolutionarily conserved stress-regulatory layer in plants. In woody species such as poplar (*Populus alba* × *P. tremula* var. *glandulosa*), dynamic m^6^A demethylation has been associated with modulation of stress-responsive pathways, including ABA signaling, ion transport, and redox-related processes under Cd exposure (Wang et al. 2025a, b). In tomato (*Solanum lycopersicum*), transcriptome-wide profiling revealed that Cd stress reshapes the m^6^A methylome, with hypomethylation frequently correlating with enhanced transcript abundance and translational efficiency of genes involved in glutathione metabolism, phenylpropanoid biosynthesis, and metal transport systems (Liu et al. 2025a, b). In alfalfa (*Medicago sativa*), coordinated alterations in DNA methylation (5mC) and m^6^A levels were observed under Cd stress, with m^6^A enrichment positively associated with gene expression of metal transporter candidates that contribute to Cd tolerance (Chen et al. 2025). Together, these findings across phylogenetically distant species—including woody plants, solanaceous crops, and forage legumes—support the notion that m^6^A-dependent regulation represents a conserved and flexible epigenetic mechanism underlying Cd stress adaptation. Therefore, the soybean-based PME strategy within the NRT1 context (Wang and Fang 2024) likely exploits a regulatory framework that might be broadly conserved across different plant species rather than representing a strictly legume-specific aspect, although further validation and investigation of specific NRT1 targets across diverse plant systems and varied experimental conditions is promising and relevant.

It is evident that NRT1 functions as an important component in the plant’s response to Cd stress by modulating nitrate reallocation and influencing Cd uptake and accumulation. While significant progress has been made in identifying key players such as NRT1.8, NRT1.5, and NRT1.1, a deeper understanding of the underlying molecular mechanisms and regulatory networks is still required. Future research should prioritize dissecting the downstream consequences of NRT1-mediated nitrate fluxes under Cd stress—for instance, by investigating their influence on Cd detoxification genes, antioxidant systems, and Cd compartmentalization.

Of worthy mention, it was also observed that nitrate supply leads to higher Cd concentrations in *A. thaliana* plants compared to ammonium treatment, highlighting the close relationship between nitrogen nutrition and Cd accumulation (more details in the literature reviewed by Guan et al. 2021). It was reported that the contribution of nitrate transporters such as NRT1.1 and NRT2.1 to Cd uptake is strictly dependent on the presence of nitrate, as differences in Cd accumulation between wild-type plants and *nrt* mutants were not observed under exclusive ammonium supply. This indicates that, considering some experiment conditions, Cd entry mediated by these transporters is coupled to NO₃⁻ uptake rather than NH_4_^+^ (Guan et al. 2021). Furthermore, considering the NRT1 research context, mutants with loss of function in nitrate transporters NRT1.1 or NRT2.1 exhibited lower Cd concentrations under low-nitrate conditions, and NRT2.1 was identified as the major contributor to Cd uptake via nitrate uptake, as its inhibition resulted in the lowest Cd levels (Guan et al. 2021). Altogether, this set of information adds nuance to previous emphasis on NRT1 family members under normal or differential nitrate conditions and suggests that different transporters may play more significant roles depending on environmental nutrient availability.

At the physiological level, nitrate (NO_3_^−^) and ammonium (NH_4_^+^) represent the two major inorganic N sources in plants and strongly influence ion balance and rhizosphere chemistry. While NH_4_^+^ uptake has been associated with proton extrusion and rhizosphere acidification, potentially altering the availability of toxic metals such as Cd, NO_3_^−^ supply has been linked to anion predominance over cations and activation of nitrate-associated transport pathways. Notably, within the experimental framework involving *Carpobrotus rossii* and *Solanum nigrum*, NH_4_^+^ nutrition resulted in greater shoot Cd accumulation relative to NO_3_^−^ supply (Cheng et al. 2016). These findings indicate that the effects of nitrogen form on Cd uptake are species-specific and depend on the experimental conditions applied. Other authors have further highlighted that Cd uptake can still be substantial under ammonium fertilization in field conditions. Either way, nitrate transport across the plasma membrane transiently polarizes and hyperpolarizes the root cell membrane, enhancing Cd uptake (Yang et al. 2020). Membrane hyperpolarization increases the electrical driving force for passive or channel-mediated Cd^2⁺^ influx (Yang et al. 2020). The majority of absorbed nitrate is translocated to the shoots, where it is either reduced by nitrate reductase or temporarily stored in vacuoles. This vacuolar sequestration is mediated by proton-coupled transport systems powered by tonoplast H^+^-ATPase and H^+^-PPase (Jian et al. 2019). Therefore, in this context, although NO_3_^−^ generally promotes greater Cd accumulation than other nitrogen forms, investigations comparing NO_3_^−^ and NH_4_^+^ found that ammonium increased rhizosphere-extractable Cd but resulted in the lowest total Cd uptake by plants, whereas nitrate treatment led to the highest Cd accumulation despite lower extractable Cd in the rhizosphere (Yang et al. 2020). These findings indicate that Cd uptake is not determined solely by rhizosphere pH or metal bioavailability. Beyond modulating such factors, nitrogen form influences Cd uptake through additional mechanisms, including alterations in root morphology, plant growth, and root cell membrane potential—the electrochemical driving force for cation uptake (Cheng et al. 2016). Indeed, nitrogen-driven regulation of gene expression and transporter activity is the primary mechanism controlling Cd absorption and accumulation in plants (Yang et al. 2020). Together, these findings suggest that the form of nitrogen supply modulates Cd uptake through both transporter-dependent and physicochemical mechanisms, highlighting nitrogen management as a potential strategy to manage Cd accumulation in crops.

## An integrative model of the NRT1 regulatory network in cadmium mitigation

Although individual NRT1 isoforms display distinct functional properties, they operate within a coordinated regulatory network that balances nutrient distribution with stress adaptation. This systemic organization can be conceptualized within the above-mentioned SINAR framework, in which transporters function not in isolation but as components of a multi-layered signaling architecture. Within this model, NRT1.1 acts as a primary nitrate sensor that triggers downstream signaling cascades involving regulators such as NRG2, ultimately coordinating the reciprocal regulation of NRT1.5 and NRT1.8. This integrated regulation—characterized by reduced xylem loading through *NRT1.5* downregulation and enhanced xylem unloading via *NRT1.8* upregulation—modulates nitrate retention in roots. Such redistribution represents a key physiological strategy that contributes to Cd detoxification and limits metal translocation to aerial tissues.

Importantly, as highlighted in the previous sections, this regulatory network is further refined by hormonal signaling pathways, including ABA, ET, and JA, which modulate transporter activity in response to environmental stress cues. Integrating these plant hormone-related findings, the regulation of SINAR under Cd stress involves a multi-hormonal network, in which the ET/JA-NRT signaling module acts as an important coordinator of responses related to NRT1.5 and NRT1.8. While ET/JA signaling is responsible for the simultaneous upregulation of *NRT1.8* and downregulation of *NRT1.5*, ABA signaling might serve as a complementary regulatory layer by specifically reinforcing the repression of NRT1.5. This convergence of ET, JA, and ABA pathways on the inhibition of NRT1 modulation highlights a robust hormonal strategy to prioritize root nitrate retention, thereby enhancing Cd tolerance through a highly integrated stress-adaptation framework. Future research might investigate if the ET/JA-NRT signaling module serves as a universal stress-adaptation framework in economically relevant crops, as current models are predominantly based on *A. thaliana*. A critical frontier lies in identifying the precise signaling molecules—whether nitrate itself or downstream assimilates like glutamate or glutamine—that perceive root nitrate status to modulate the growth-tolerance trade-off. In addition, further research might also explore high-precision strategies to uncouple Cd tolerance from growth inhibition, potentially related to ABA and ET/JA-mediated aspects, while maintaining active SINAR-driven nitrate reallocation to roots. Furthermore, the NRT1.2/AIT1 module exemplifies this integration by linking nitrate transport with ABA-mediated regulation of metal uptake, particularly through modulation of IRT1-related aspects. This interaction adds an additional layer of systemic control, reinforcing modulation towards Cd entry and accumulation.

**Fig. 1:**
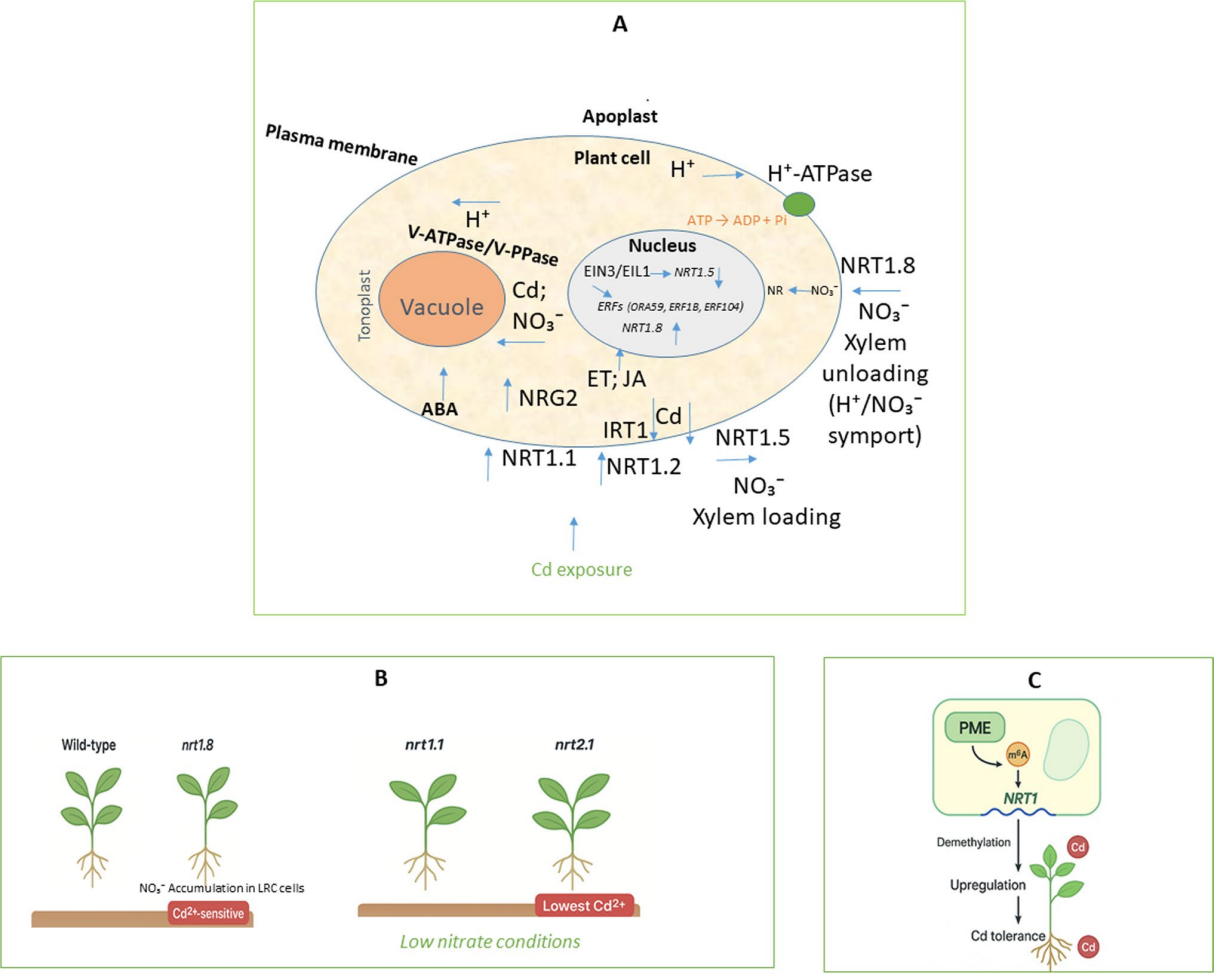
NRT1-mediated nitrate reallocation and its regulatory network in plant adaptation to Cd stress: This schematic and integrated model conceptually illustrates the complex roles of members of the NRT1 nitrate transporter family and their regulatory network in modulating nitrate distribution and plant adaptation to Cd stress. For schematic simplification and to facilitate visualization of the integrated model, vascular-associated cells are represented generically. **A** Integrated model at the cell level: *NRT1.8*, predominantly expressed in xylem parenchyma cells, mediates nitrate unloading from the xylem sap. Its expression is strongly upregulated in roots under Cd stress, leading to enhanced nitrate accumulation in this organ, which is essential for mitigating Cd toxicity and reducing Cd translocation to shoots. Plasma membrane H^+^-ATPases generate the electrochemical gradient by extruding protons into the apoplast. This gradient drives the proton-coupled nitrate unloading mediated by NRT1.8 (H^+^/NO_3_^−^ symport), enabling nitrate retrieval from the xylem into parenchyma cells. In contrast, NRT1.5 facilitates nitrate loading into the xylem, a process downregulated by Cd and ABA signaling, favoring root nitrate retention. Vacuolar sequestration of Cd and nitrate depends on tonoplast energization driven by V-ATPase and V-PPase activity. Proton translocation into the vacuole establishes the electrochemical gradient required for secondary transport processes that facilitate ion compartmentalization. The coordinated yet contrasting regulation of *NRT1.5* and *NRT1.8* under heavy metal stress promotes the reallocation of nitrate to the roots, a key mechanism of tolerance. NRT1.1 functions as a nitrate sensor that induces the expression of *NRG2*, which acts as a downstream regulator. This signaling module coordinates nitrate allocation by repressing *NRT1.5* expression (reducing xylem loading) and upregulating *NRT1.8* expression (enhancing xylem unloading). The resulting nitrate reallocation to roots facilitates Cd sequestration into root vacuoles, enhancing plant tolerance. Furthermore, NRT1.2 (also known as AIT1) acts as a dual nitrate and ABA importer located at the root plasma membrane. Under Cd exposure, NRT1.2-mediated ABA import into root cells might trigger the downregulation of *IRT1*, reducing Cd uptake, and also its accumulation in plant organs. Cd stress also triggers ET and JA signaling, which converge at the EIN3/EIL1 transcription factors. EIN3 directly represses *NRT1.5* to reduce xylem loading, while simultaneously inducing ERFs, such as ORA59, to upregulate *NRT1.8* for enhanced xylem unloading. This ET/JA-NRT module coordinates Stress-Initiated Nitrate Allocation to Roots (SINAR), modulating Cd tolerance at the expense of regular plant growth through a nitrate reductase-dependent mechanism. Arrows indicate either transport directions (e.g., ion fluxes across membranes) or regulatory interactions, including activation/upregulation and repression/downregulation, according to the specific context of each module and the evidence within the contextual framework presented in the respective sections of this article. **B** *nrt1.8* mutants show nitrate-dependent Cd sensitivity and NO_3_^−^ accumulation in the LRC cells, while NRT2.1 is the main contributor to Cd uptake under low-nitrate conditions, compared to NRT1.1. **C)** In the context of epigenetic modulation: targeted m^6^A (N6-methyladenosine) demethylation of NRT1 enhances its expression and modulates Cd tolerance. Panels **A**–**C** illustrate the information derived from the studies referenced in the previous sections. **Symbols and abbreviations**: *ABA* abscisic acid, *Cd* cadmium, *ERF104* ethylene response factor 104, *ERF1B* ethylene response factor 1B, *ERFs* ethylene response factors, *EIN3/EIL1* ETHYLENE INSENSITIVE3/EIN3-LIKE1, *ET* ethylene, *IRT1* Iron-Regulated Transporter 1, *JA* jasmonic acid, *LRC* lateral root cap, *m6A* N6-methyladenosine, *NRT1* Nitrate Transporter1, *NRG2* Nitrate Regulatory Gene 2, *NR* nitrate reductase, *NO*_*3*_^*−*^ nitrate ion, *ORA59* Octadecanoid-Responsive *Arabidopsis* AP2/ERF59, *PME* plant m^6^A editor. Arrows indicate either transport directions or regulatory interactions, specifically upregulation or induction, which reflect an increase in gene expression or activity, and downregulation or suppression, which reflect a decrease in gene expression or activity

The cohesive nature of this model is embedded throughout the article through a structured, multi-level synthesis. Tables 1 and 2 provide an overview of representative functional roles and the corresponding experimental evidence for individual NRT1 isoforms across different plant species, thereby delineating the core components of the regulatory network. Figure 1 presents a schematic synthesis of the key mechanisms discussed in the previous sections, highlighting the roles of NRT1 transporters in nitrate redistribution and Cd detoxification. By integrating the functional evidence summarized in the tables with the spatial and regulatory interactions illustrated in Fig. 1, a mechanistic and integrated framework highlighting how the NRT1 family coordinates nitrogen metabolism with plant stress adaptation under Cd exposure is provided.

## Advancing perspectives in NRT1–cadmium research

### From model systems to crops and diverse plant species: integrating multiple approaches

To further advance the understanding of NRT1-mediated responses to Cd, future research might expand beyond traditional model systems to include a broader range of agronomically relevant species and complex environmental scenarios, in addition to the crops and plant species already highlighted in the previous sections. Recent omic and functional studies have already begun to broaden the NRT1 landscape, identifying extensive gene families and tissue-specific expression patterns in crops such as Chinese cabbage (Zhang et al. 2023), oat (Cheng et al. 2025), and dwarf coconut (Liu et al. 2025a, b). Besides maintaining growth under limited nitrogen availability through high-affinity transport (Ye et al. 2019), the investigations in multiple species highlight the versatility of NRT1 members across diverse taxa, including their roles in responding to cold stress in woody species (Liu et al. 2025a, b). Furthermore, experimental configurations involving other heavy metals, such as zinc toxicity in canola, have demonstrated that the downregulation of *NRT1.5* is a common response to metal stress, which can be mitigated by signaling molecules like putrescine (Aghajanzadeh et al. 2025). The integration of advanced agricultural techniques, such as rootstock grafting in cucumbers, also reveals that the upregulation of *NRT1.2* and *NRT1.5* can enhance nitrogen-use efficiency and stress resilience (Wang et al. 2025a, b), while the specific ratio of inorganic nitrogen sources further modulates the leading role of isoforms like *NRT1.1* and *NRT1.7* in nutrient transport (Shi et al. 2025). Despite these insights, several studies have utilized controlled, single-stress environments. In field conditions, however, plants are simultaneously subjected to multiple stressors—including heavy metal contamination, temperature fluctuations, and varying nutrient levels—that likely trigger synergistic or antagonistic regulatory responses within the NRT1 system. Therefore, future research might focus on characterizing the NRT1-Cd intersection in diverse crop species under multi-stress field conditions to develop robust strategies for improving both food safety and mitigation-related aspects.

Considering this cross-species focus, future research might also consider the promising direction involving the engineering of NRT1 transporter variants with reduced Cd permeability while preserving efficient nitrate uptake, thereby enhancing tolerance without compromising nutrient acquisition. Achieving this goal will require detailed characterization of the molecular and structural determinants governing substrate specificity in NRT1.1 and other family members, establishing a foundation for rational protein design and precision engineering. In parallel, the potential of exogenous regulators, including plant growth regulators such as ABA, ET and JA, might be systematically evaluated to determine their capacity to modulate NRT1 activity and improve Cd resilience under realistic agronomic conditions, with careful consideration of efficacy, safety, and long-term impacts. Expanding investigations into the conservation of NRT1 regulatory networks across diverse crops exposed to Cd may further reveal evolutionary trade-offs affecting growth, development, and nutrient homeostasis, particularly under multi-stress field environments. Clarifying isoform-specific contributions remains equally critical, as distinct NRT1 members likely exert differential control over Cd uptake, redistribution, and tolerance depending on species and environmental context. Precision manipulation of expression levels or transporter activity could help minimize Cd accumulation in edible tissues while safeguarding yield and nutritional quality, thereby directly addressing food safety concerns. Moreover, dissecting the interactions between NRT1 transporters and other heavy metal transport systems will be essential to understand how coordinated or competing pathways shape overall metal homeostasis and Cd partitioning within the plant.

Some authors have pointed out that nitrogen acquisition in plants is regulated by multiple transporters. This framework suggests that when one nitrogen uptake pathway is inhibited or blocked, other transport systems may compensate, maintaining overall nutrient acquisition (Guan et al. 2015). This redundancy likely explains why the inhibition of NRT1.1-mediated nitrate uptake only caused a modest impact on plant growth in either standard growth conditions in some experiments (Mao et al. 2014). Consequently, biotechnological strategies aimed at modifying nitrate uptake pathways to reduce cadmium accumulation in crops may offer a dual benefit: they may lower both the risks associated with Cd exposure and the challenges of nitrogen fertilizer management, while exerting minimal negative effects on crop productivity (Guan et al. 2015). Further research might focus on exploring the regulatory networks controlling transporter redundancy, which may provide novel targets for breeding or engineering crops with enhanced resilience to Cd stress and optimized nutrient status.

Beyond these targeted approaches, integrative multi-omics strategies provide an additional and indispensable layer of resolution. In addition to the studies discussed throughout this article, our research group has contributed to the plant Cd field by investigating organ- and tissue-specific responses through combined and multiple omic analyses, together with grafting-based experimental systems (Marques et al. 2021, 2023a, b, 2024). These efforts highlight that Cd tolerance and accumulation are spatially regulated processes involving coordinated inter-organ communication and post-translational modulation, reinforcing the need to interpret NRT1-mediated mechanisms within a dynamic, systems-level framework. Integrating structural biology, functional genetics, hormone signaling, transporter interaction networks, and multi-omic analyses will be pivotal for developing and incorporating more robust strategies that enhance Cd tolerance and mitigation while sustaining plant growth and agricultural productivity related to the relevant NRT1-related aspects.

### Precision engineering: tissue-specific promoters and targeted strategies

Recent advances in plant biotechnology highlight the need to move beyond constitutive overexpression strategies when targeting metal transport and detoxification pathways. Although constitutive promoters are widely used to drive strong and stable gene expression, they activate transgenes across all tissues and developmental stages, often resulting in unnecessary metabolic costs and reduced regulatory precision. In the context of Cd management, the development and application of tissue-specific promoters—especially those with strong root-preferential activity—represent a more refined alternative. By directing gene expression specifically to roots, recent studies provide compelling evidence that promoter selection can effectively restrict Cd translocation or reducing Cd accumulation in shoots while preserving plant performance. For example, in rice, Zhao et al. (2025) engineered lines expressing *OsHMA3* under the control of the *OsYSL16* promoter, achieving enhanced Cd sequestration in root vacuoles and markedly reduced Cd translocation from roots to shoots and grains, without penalties to yield or micronutrient accumulation. Similarly, Cai et al. (2019) demonstrated that root-specific expression of *OsHMA3* in tobacco, driven by the *NtREL1* promoter, significantly limited Cd movement to aerial tissues while maintaining normal biomass production. Sun et al. (2025) identified the root-specific promoter *PRSEP7* in poplar and showed that *PRSEP7*-driven expression increased Cd retention in roots and decreased shoot accumulation, reinforcing the feasibility of spatially controlled detoxification strategies; notably, several root-specific genes identified in that study were associated with nitrate and ammonium transport, underscoring the tight integration between nitrogen transport pathways and metal homeostasis (Sun et al. 2025). Together, these findings support precision promoter engineering as a viable strategy to confine Cd belowground.

Future studies exploring NRT1-associated regulatory networks and nitrate-dependent signaling as additional targets to uncouple Cd accumulation from nitrate distribution to shoots may provide a mechanistic framework for developing crops with reduced Cd translocation without compromising nitrogen-use efficiency or shoot and whole-plant nutritional status. From a mechanistic standpoint, Cd enters root cells largely through transport systems that primarily mediate the uptake of essential metals. Complete disruption of these transporters may reduce Cd accumulation, but it also risks impairing the acquisition of nutrients required for normal growth. It has been suggested that, in certain plant systems, a more strategic approach involves limiting Cd xylem loading rather than blocking its initial uptake by enhancing Cd sequestration within root vacuoles, for example, which may effectively retain Cd belowground, thereby reducing its translocation to edible tissues while avoiding penalties to nutrient status and overall plant performance (Cai et al. 2019). This concept aligns with proposals that root-targeted expression of genes involved in metal chelation, transport, or compartmentalization could improve detoxification efficiency while minimizing systemic and nutritional side effects. Moreover, as we recently highlighted, co-expression strategies can further enhance or refine the modulation of Cd detoxification capacity by simultaneously activating complementary pathways related to chelation and vacuolar transport (Marques et al. 2025b; Marques and Mason 2025).

A complementary perspective involves the systematic identification of *cis*-regulatory elements and tissue-specific promoters controlling Cd uptake, transport, and detoxification, providing a molecular toolkit for fine-tuned genetic strategies to reduce Cd accumulation (Xu et al. 2024). Such coordinated regulation might increase the efficiency of metal immobilization without compromising plant growth or essential nutrient homeostasis. These findings support a precision-engineering framework in which spatially controlled and combinatorial gene expression might limit Cd translocation while preserving nitrogen transport and overall plant fitness.

### Bibliometric trends in NRT1-related cadmium research

**Fig. 2:**
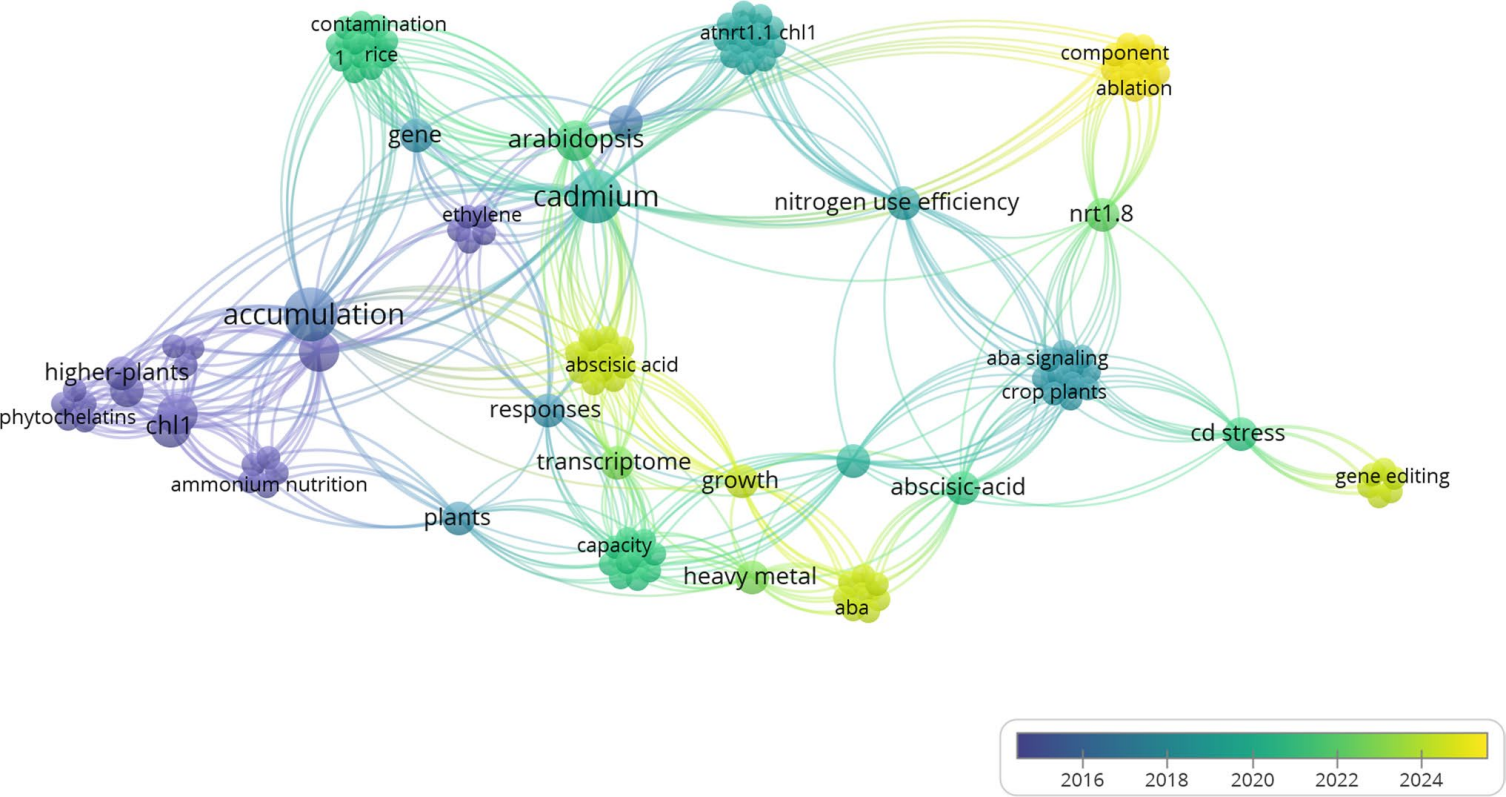
Bibliometric analysis of the research landscape on NRT1 transporters and Cd exposure. The network map illustrates the co-occurrence of author keywords extracted from scientific literature. Nodes (spheres) represent specific research terms, and their size is proportional to the frequency of keyword occurrence within the dataset. The lines (links) between nodes indicate the strength of co-occurrence relationships, reflecting how frequently terms appear together in the same publications. The color gradient represents the average publication year

To complement this perspective, we performed a bibliometric analysis using an international database, offering an integrated overview of scientific themes and emerging focal points in NRT1-related Cd research. The related methodology is provided in Supplementary Material S1. Utilizing VOSviewer software (v. 1.6.20; Leiden University, The Netherlands) (van Eck and Waltman 2010), we systematically analyzed the retrieved literature to generate a keyword co-occurrence network map (Fig. 2), which highlights the evolution of the field from fundamental nitrogen uptake to sophisticated biotechnological interventions. The integration of bibliometric data from Fig. 2 with the functional findings summarized in Tables 1 and 2 and Fig. 1 highlights a significant shift in research priorities over the last decade. Early research was related to the foundational characterization of NRT1.1 transporter, phytochelatins, and basic accumulation patterns in model species like *A. thaliana*. However, the transition toward green and yellow nodes signifies a modern frontier where the focus has moved toward hormonal signaling (e.g., ABA), transcriptome-wide responses, and high-precision tools such as gene editing. This evolution suggests that future research is increasingly tasked with moving beyond descriptive accumulation studies toward the active manipulation of regulatory hubs to enhance nitrogen use efficiency while simultaneously mitigating Cd toxicity.

Another striking innovation observed in the bibliometric map is the recent prominence of “gene editing” and “abscisic acid” as current hotspots (yellow nodes). While the information provided in the previous sections details specific experimental evidence of ABA-mediated downregulation of *NRT1.5* and induction of *NRT1.2* (AIT1), for instance, the network map confirms that this hormonal intersection may now be a relevant pillar for future plant improvement strategies. A critical perspective emerging from this analysis is the potential to use CRISPR/Cas9-based gene editing to fine-tune the expression of specific NRT1 isoforms—such as NRT1.8—to optimize stress-initiated nitrate allocation to roots without the typical growth penalties associated with constitutive stress responses. Furthermore, the proximity of the “transcriptome” node to “growth” and “responses” in the map highlights the necessity of a systems-level understanding. As emphasized by our research group’s recent work in phosphoproteomics and multi-omics (Marques et al. 2021, 2023, 2024), these large-scale datasets allow for the identification of the specific phosphorylation events and post-transcriptional modifications that govern transporter-related patterns and activity under Cd stress.

The synergy between the bibliometric trends in Fig. 2 and the functional data in Tables 1 and 2 and Fig. 1 underscores that the next decade of research might prioritize the unlinking of Cd accumulation from nitrate distribution. By leveraging the emerging focus on tissue-specific promoters and targeted mRNA demethylation (also involving PMEs), researchers might now aim to confine Cd belowground in root vacuoles while maintaining robust nitrogen transport to the shoots. This integrated approach—combining bibliometric foresight with molecular precision—will be relevant for developing the next-generation of crops and other groups of relevant plant species ensuring Cd mitigation in heavy metal-contaminated environments.

## Concluding remarks and additional future directions

The information presented and discussed throughout this article highlights the multifaceted roles of NRT1 transporters related to the modulation of plant responses to Cd exposure, encompassing aspects of nutrient status in addition to nitrogen and metal accumulation and uptake, plant signaling, root uptake, isoform-specific regulation, and coordinated nutrient and metal homeostasis. Toward this integrative perspective, targeted biotechnological and engineering strategies directed at NRT1 transporters hold considerable promise for modulating Cd tolerance while preserving agronomic performance and the nutritional and physiological quality of crops and relevant plant species. Approaches may include the selection of strategic tissue-specific promoters and the fine-tuning of transporter expression or substrate specificity through innovative tools such as m6A-based regulatory editing and PME-related strategies. Substrate selectivity, in particular, might be refined through complementary strategies including targeted amino acid substitutions informed by structural modeling, rational protein design, and directed evolution. Transgenic overexpression or silencing of carefully selected NRT1 variants in defined tissues may further enhance nitrate acquisition while limiting Cd accumulation in edible organs. Likewise, CRISPR/Cas-mediated editing of promoters or *cis*-regulatory elements enables precise modulation of expression patterns in tissues directly involved in Cd uptake and translocation, recognizing that functional outcomes are strongly dependent on cellular and developmental context. Directed evolution coupled with high-throughput mutagenesis in heterologous or cell-based systems represents an additional avenue which might generate NRT variants with altered substrate properties prior to plant implementation. Crucially, translating these advances from model species to agriculturally important crops—particularly those prone to significant Cd uptake and accumulation in relevant plant tissues and organs—is essential for limiting Cd entry into the food chain, improving overall metal management, and strengthening crop resilience in contaminated soils. Ultimately, a comprehensive understanding of NRT1 functions, together with insights into nitrogen availability, nutritional status, and hormonal signaling, provides a strategic foundation for developing integrated mitigation approaches capable of sustaining productivity and ensuring overall Cd mitigation in addition to safer food production under Cd-exposed environments.

## Supplementary Information

Below is the link to the electronic supplementary material.

Supplementary file1 (DOCX 14 KB)

## Data Availability

This manuscript has no associated data.
