# Supplementary material for: Frontiers in cadmium mitigation: harnessing Nitrate Transporter 1 (NRT1) for plant systems
Source: Planta. 2026 Mar 4;263(4):89. doi: 10.1007/s00425-026-04963-7 (PMC12957033; doi:10.1007/s00425-026-04963-7)
Supplement: Supplementary file 1 — Supplementary file1 (DOCX 14 KB) [file 425_2026_4963_MOESM1_ESM.docx]

**Supplementary file 1**

Frontiers in Cadmium Mitigation: Harnessing Nitrate Transporter 1 (NRT1) for Plant Systems

Deyvid Novaes Marques ^1^*, Ricardo Antunes Azevedo^1^

^1^ Department of Genetics, Luiz de Queiroz College of Agriculture (ESALQ), University of São Paulo (USP), Piracicaba, São Paulo (SP), Brazil

***** Correspondence: deyvidnovaes@gmail.com

**Bibliometric and Experimental Screening of NRT1-Related Studies in Cadmium-Exposed Plants**

A structured bibliographic search was conducted in the Web of Science Core Collection to identify original research articles addressing the relationship between NRT1 transporters and cadmium (Cd) exposure in plants. The search was performed using the query “(cadmium OR Cd OR heavy metal)” in the Abstract field combined with “NRT1” in the Abstract field. To ensure thematic precision and methodological rigor, all retrieved documents were subjected to detailed manual screening.

Only original experimental research articles were retained. Review articles, meta-analyses, opinion papers, and purely descriptive transcriptomic surveys lacking functional validation were excluded. Studies were also removed when NRT1 was mentioned only peripherally, without direct experimental investigation under cadmium exposure. Additionally, publications that addressed heavy metals in general but did not experimentally evaluate cadmium were excluded. This curation process ensured that the final dataset comprised studies presenting new experimental evidence linking NRT1-family transporters to Cd-related physiological, molecular, or genetic responses in plants.

The curated and filtered records were subsequently exported from the Web of Science Core Collection and analyzed using VOSviewer (version 1.6.20, Leiden University, The Netherlands) (van Eck, 2010). VOSviewer was used to generate the bibliometric map based on keyword co-occurrence analysis and to identify conceptual clusters within the dataset, highlighting thematic connections between NRT1-related research and Cd exposure in plants.

**Reference**

van Eck, N.J.; Waltman, L. Software survey: VOSviewer, a computer program for bibliometric mapping. Scientometrics 2010, 84(2), 523-538. <https://doi.org/10.1007/s11192-009-0146-3>
